# Supplementary material for: A Consolidated Saccharification, Fermentation, and Transesterification Process (cSFT) Converting Castor Oil to Biodiesel with Cellulose-Derived Ethanol
Source: Int J Mol Sci. 2025 Dec 10;26(24):11902. doi: 10.3390/ijms262411902 (PMC12732804; doi:10.3390/ijms262411902)
Supplement: Supplementary file 1 [file ijms-26-11902-s001.zip › ijms-4007729-supplementary.pdf]

Supplementary Data

A consolidated saccharification, fermentation and transesterification process (cSFT) for "one-pot" conversion of cellulose to biodiesel

Ester Korkus Hamal[1], Gilad Alfassi[2], Dmitry M. Rein[1], Yachin Cohen[1]*

[1]Department of Chemical Engineering, Technion - Israel Institute of Technology, Haifa 3200003, Israel

[2] Department of Biotechnology Engineering, Braude College of Engineering, Karmiel 2161002, Israel.

* Corresponding author. E-mail address: yachinc@technion.ac.il

The dimensions of cellulose-coated micro-particles (Table S1) evaluated by analysis of 20 cryo-SEM images, using imageJ software, scientific image analysis.

Table S1. Dimensions of cellulose-coated micro-particles based on cryo-SEM images.

	Mean diameter (μm)	Diameter range (μm)	STD
Micro-particle	0.8	0.2-2.4	0.5

Light scattering (LS)

Particle size and size distribution of the cellulose-coated o/w emulsion micro-particles and yeasts were monitored by light scattering using a Mastersizer 2000 (Malvern Co. Ltd., UK), equipped with He-Ne red laser (λ=633 nm). The particle size distribution was calculated on a number-average basis and volume-average basis.

Light scattering measurements were implemented on the cellulose-coated micro-particles and yeasts. The first peak at a lower particle size (0.1-1 μm) is attributed to individual cellulose-coated micro-particles, while the peak at a larger size (1-10 μm) is attributed to individual yeast cells. The largest particle size (10-100 μm) is attributed to aggregation of cellulose-coated micro-particles and yeast cells.

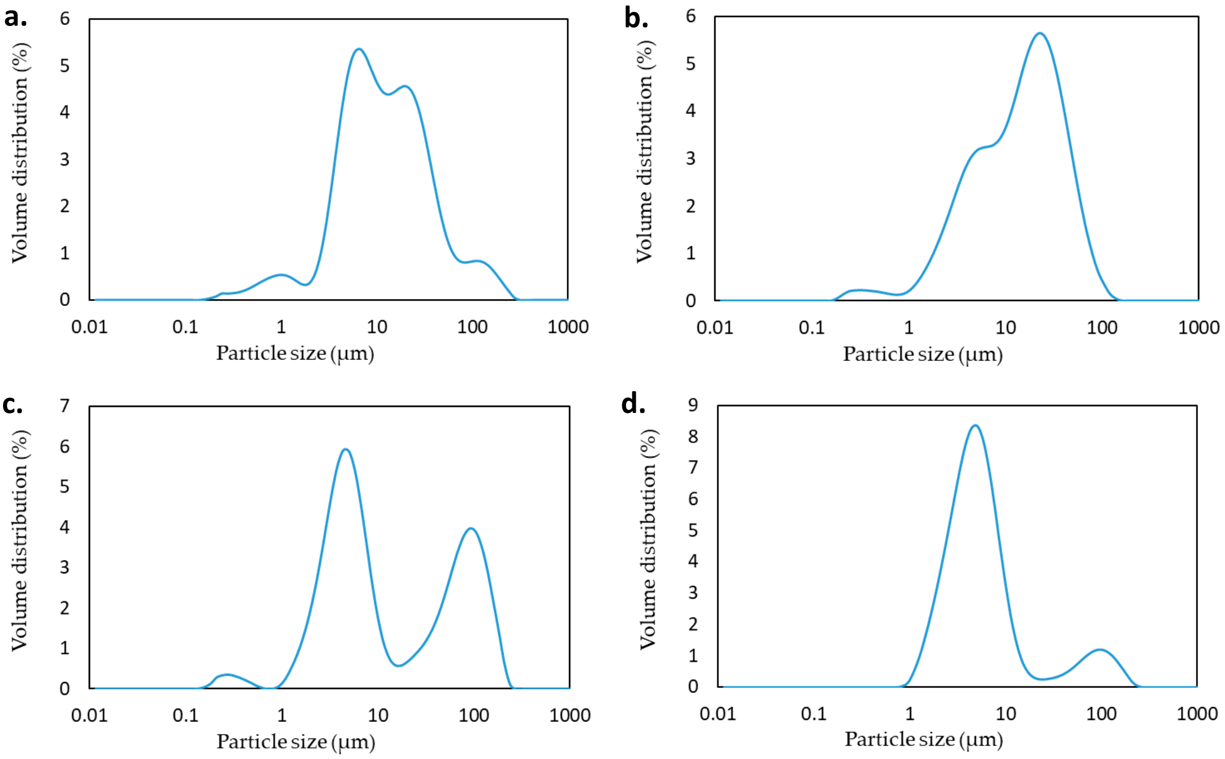

Figure S1. Particle size distributions (by volume) at: a. time 0; b. 24 hours; c. 48 hours; d. 72 hours.

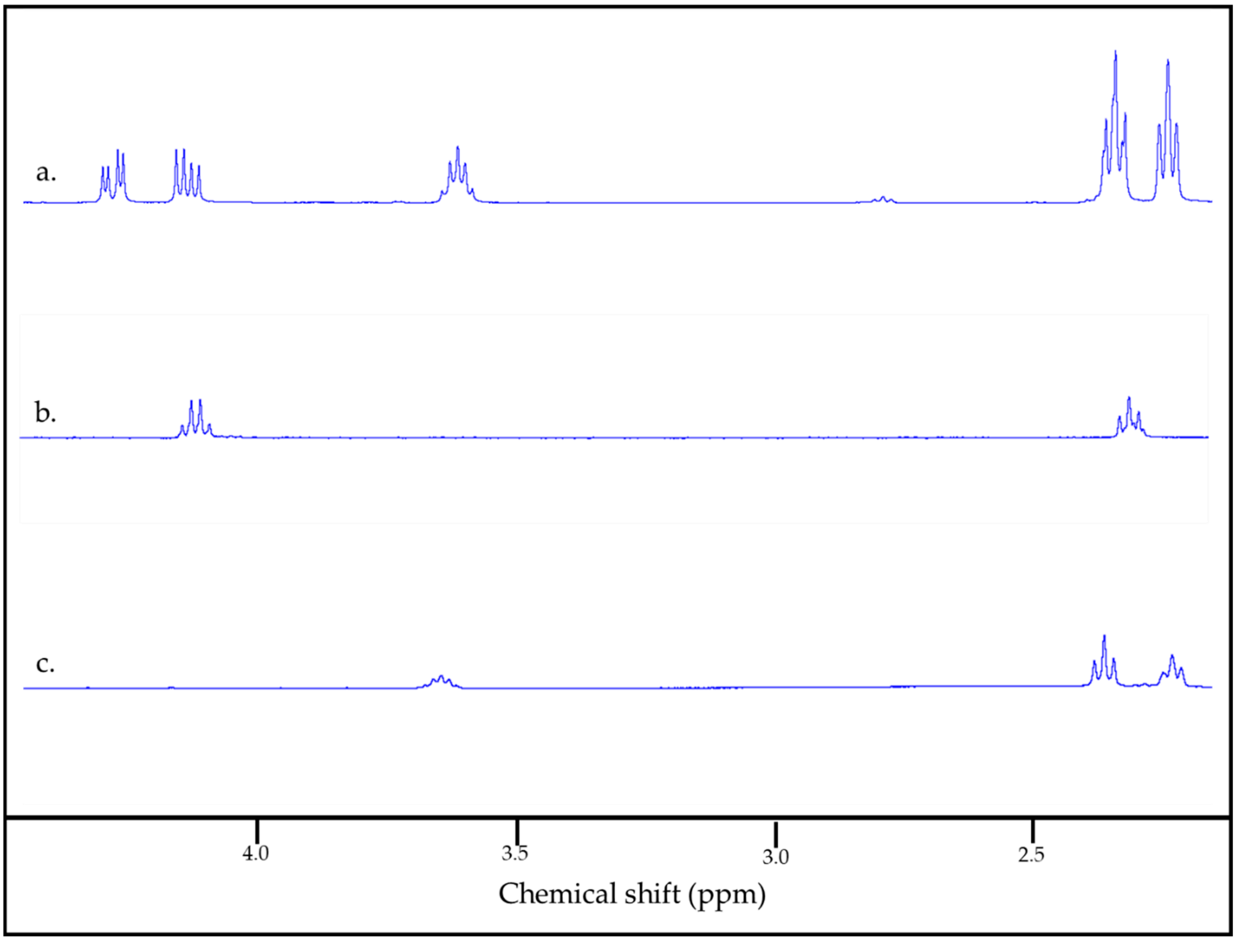

Figure S2. ^{1}H NMR spectra of: (a) castor oil (b) pure FAEE (c) FFA. As mentioned in our previous work[1]

The quantification of the percent conversion of oil to FAEE using ^{1}H NMR spectroscopy was done by using equation S1 proposed by Sumit.[2]

$$\%C_{EE} = 100 \times \frac{I_{TAG+EE} - I_{TAG}}{I_{\alpha CH_2(TAG+EE+FFA)} + 2I_{TAG}} \qquad (S1)$$

$\%C_{EE}$ is the percent conversion of oil to FAEE. I_{TAG+EE} is the joint integration in the range 4.10-4.17 ppm (glyceryl methylenic hydrogen of oil and ethoxy hydrogen of FAEE, marked with red dot). I_{TAG} is the integration in the range of 4.28-4.32 ppm (glyceryl methylenic hydrogen of oil, marked with red dot) and $I_{TAG+EE+FFA}$ is the joint integration in the range of 2.24-2.38 (α-acyl methylenic hydrogen in oil, FFA and FAEE, marked with a green dot).

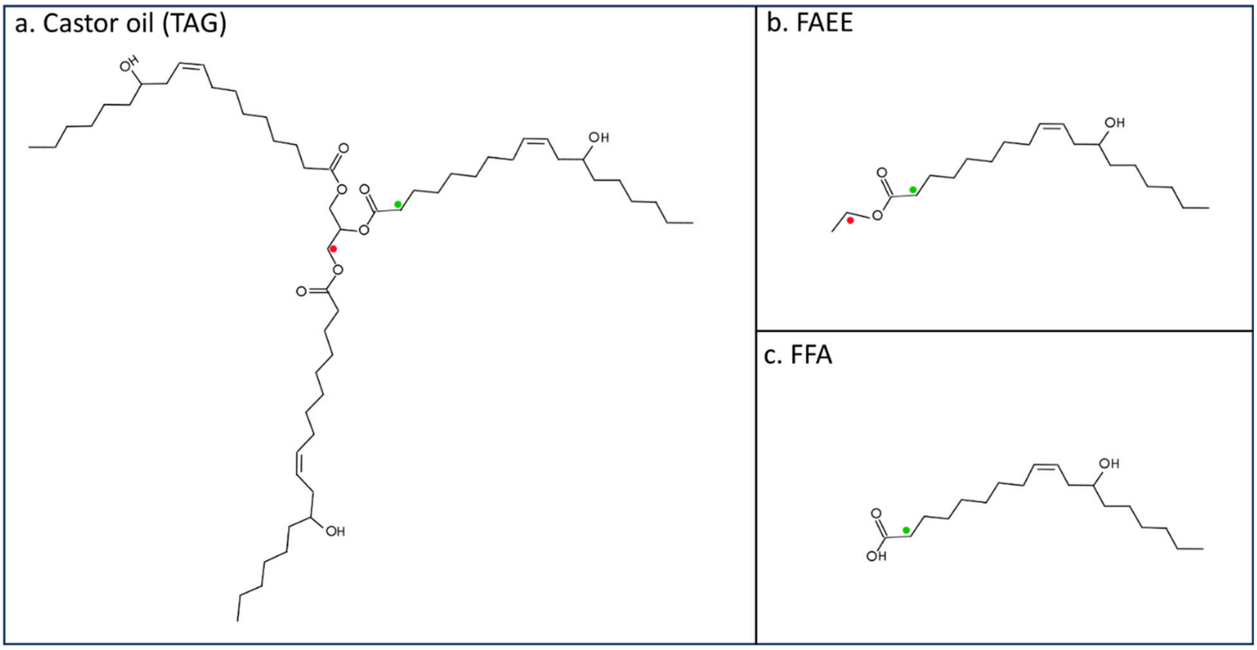

Figure S3. a. TAG (castor oil); b. FAEE; C. FFA. The green and red dots represent the α-acyl methylenic hydrogen and the glyceryl methylenic hydrogen, respectively.

References

1. Korkus Hamal, E., Alfassi, G., Khalfin, R., Rein, D. & Cohen, Y. Towards one-pot consolidated bioprocessing of cellulose to biodiesel : lipase-catalyzed transesteri fi cation at cellulose-coated oil-in-water emulsions as micro-reactors. *J. Chem. Technol. Biotechnol.* (2022)
2. Jaiswal, S. K., Tejo Prakash, N. & Prakash, R. 1 H NMR Based Quantification of Ethyl Ester in Biodiesel: A Comparative Study of Product-Dependent Derivations . *Anal. Chem. Lett.* **6**, 518–525 (2016).
